# The Modification of the Gut Microbiota via Selected Specific Diets in Patients with Crohn’s Disease

**DOI:** 10.3390/nu13072125

**Published:** 2021-06-22

**Authors:** Eliza Starz, Karolina Wzorek, Marcin Folwarski, Karolina Kaźmierczak-Siedlecka, Laura Stachowska, Katarzyna Przewłócka, Ewa Stachowska, Karolina Skonieczna-Żydecka

**Affiliations:** 1 Students’ Scientific Circle of Clinical Nutrition, Medical University of Gdansk, 80-211 Gdańsk, Poland; eliza.starz@gumed.edu.pl (E.S.); wzorekka@gumed.edu.pl (K.W.); 2 Department of Clinical Nutrition and Dietetics, Medical University of Gdansk, 80-211 Gdańsk, Poland; 3 Department of Surgical Oncology, Medical University of Gdansk, 80-210 Gdansk, Poland; karolina.kazmierczak-siedlecka@gumed.edu.pl; 4 Department of Biochemical Sciences, Pomeranian Medical University in Szczecin, 71-460 Szczecin, Poland; laura.stachowska@pum.edu.pl (L.S.); karzyd@pum.edu.pl (K.S.-Ż.); 5 Department of Bioenergetics and Physiology of Exercise, Medical University of Gdansk, 80-210 Gdańsk, Poland; kprzewlocka@gumed.edu.pl; 6 Department of Human Nutrition and Metabolomics, Pomeranian Medical University in Szczecin, 71-460 Szczecin, Poland; ewa.stachowska@pum.edu.pl

**Keywords:** Crohn’s disease, elimination diets, microbiome

## Abstract

Gastrointestinal symptoms in Crohn’s disease (CD) are common and affect the quality of life of patients; consequently, a growing number of studies have been published on diet interventions in this group. The role of the gut microbiota in the pathogenesis and the progression of inflammatory bowel diseases (IBD), including CD, has been widely discussed. Mainly, a decreased abundance of Firmicutes, species of the *Bifidobacterium* genus, and the *Faecalibacterium prausnitzii* species as well as a reduced general diversity have been described. In this review article, we summarize available data on the influence of reduction diets on the microbiome of patients with CD. One of the most frequently used elimination diets in CD patients is the low-FODMAP (Fermentable Oligosaccharides, Disaccharides, Monosaccharides, and Polyols) diet. Although many papers show it may reduce abdominal pain, diarrhea, or bloating, it also reduces the intake of prebiotic substances, which can negatively affect the gut microbiota composition, decreasing the abundance of *Bifidobacterium* species and *Faecalibacterium prausnitzii*. Other elimination diets used by IBD patients, such as lactose-free or gluten-free diets, have also been shown to disturb the microbial diversity. On the other hand, CDED (Crohn’s disease exclusion diet) with partial enteral nutrition not only induces the remission of CD but also has a positive influence on the microbiota. The impact of diet interventions on the microbiota and, potentially, on the future course of the disease should be considered when nutritional guidelines for IBD patients are designed. Dietetic recommendations should be based not only on the regulation of the symptoms but also on the long-term development of the disease.

## 1. Introduction

**Figure 1:**
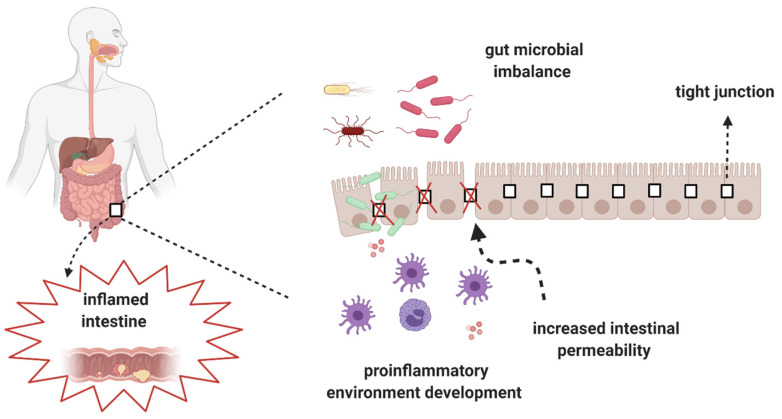
Gut microbial imbalance and development of CD.

Crohn’s disease (CD) is an inflammatory bowel disease (IBDs) that may affect every segment of the gastrointestinal tract [1,2] and cause complications, such as lesions and fistulas. The most common symptoms of CD are abdominal pain, diarrhea, rectal bleeding, unintentional weight loss, fever, and chronic fatigue [3,4]. The etiopathogenesis of CD is multifactorial and includes, among others, genetic background, environmental factors, and gut microbiota imbalance (dysbiosis). The association between gut dysbiosis and the development of CD has been recognized during the last several years. In patients with CD, gut dysbiosis and increased gut permeability are observed (Figure 1) [5].

Currently, there are therapeutic methods that modulate and restore the gut microbiota balance, such as those based on the administration of prebiotics, probiotics, and symbiotics [6]. Nevertheless, the composition and activity of the gut microbiota may also be altered via nutrition (Table 1). Restrictive diets may reduce the symptoms of CD, influencing the composition of the microbiome. These changes may have the potential to affect the course of the disease. Therefore, in this review, we concentrate on the role of selected diets (low-FODMAP, gluten-free, lactose-free, ketogenic, specific carbohydrate diet, Paleo diet, and Crohn’s disease exclusion diet) in the treatment of CD, presenting their impact on the composition as well as the activity of the gut microbiota.

## 2. Alterations of the Gut Microbiota in CD

The human microbiome varies depending on the body site and consists of bacteria, archaea, fungi, protozoa, and viruses. Three main bacterial phyla and some genera dominate in the gastrointestinal tract, i.e., Bacteroidetes 9–42%, Firmicutes 30–52%, and Acinobacteria 1–13%, and *Lactobacilli*, *Streptococci*, and *Escherichia coli* (*E.coli*), respectively [5]. Intestinal microorganisms are associated with the development and activity of IBDs, including CD. Several theories support the role of the microbiome in the pathogenesis of IBD. IBD could be due firstly, to the presence of long-lasting infections caused by pathogenic species such as *Mycobacterium avium* subspecies *paratuberculosis* or adhesion-invasive *Escherichia coli (AIEC)*; secondly, to immoderate bacterial translocation and a shift from a balanced to a dysbiotic microbiota composition [6]. On the other hand, a protective effect is observed by specific bacterial metabolites such as Short-chain fatty acids (SCFAs), which reduce local inflammation [7]. A study by Imhann showed alterations of the gut microbiome (mainly, lower abundance of the *Roseburia* genus) in healthy individuals with a high genetic risk of IBD [8]. In a recent meta-analysis, Paramsothy showed that fecal microbiota transplantation (FMT) has a positive effect on remission induction in ulcerative colitis (UC) patients. It was shown that 50.5% of CD patients achieved remission; however, the studies were heterogeneous, and future well-designed randomized trials are needed to confirm the validity of this method for CD treatment [1]. The intestinal microbiota in CD is characterized by a decrease in the diversity and total number of microorganisms of up to 50% compared to healthy individuals [2,3]. Moreover, a reduced abundance of Firmicutes is noticed, which is associated with a parallel increase in Proteobacteria [4]. Of the Firmicutes phylum, the less common species in IBD are *Clostridium leptum* and *Feacalibacterium prausnitzii* [2,9]. Commensal bacteria belonging to *Clostridium* cluster IVa are also characterized by reduced abundance [10]. A significant relative decrease of *Clostridium leptum* and *Bifidobacteria* from the Bifidobacteriaceae family and the Lachnospiraceae family has been observed in patients with CD compared to healthy subjects [11]. On the other hand, studies showed an increased number of Actinobacteria, Proteobacteria [2,12,13,14], and Enterobacteriaceae both in remission and in active CD [4]. Potentially unfavorable and proinflammatory intestinal microorganisms such as *Raminococcus gnavus* [10], *Clostridium difficile*, *Escherichia coli*, *Campylobacter concisus* [15,16], and *Shigella flexneri* [17] are present in increased numbers in comparison to healthy controls, shortening the remission of the disease [18]. A high abundance of the specific *Escherichia coli (AIEC)* strain *LF82* has been demonstrated to be associated with ileal CD. AIEC strains were isolated from almost one-third of ileal specimens in CD as compared with 6% in ileal controls and less than 5% in colonic samples from both IBD patients and controls [19]. Colonization by this bacterium caused intestinal inflammation, increased the expression of proinflammatory cytokines, and stimulated the function of T-helper 17 lymphocytes (Th17) cells in vivo [20,21]. The prevalence of this invasive strain was associated with inflammation of the ileum but not of the colon [19]. Th17 produce the proinflammatory cytokine interleukin -23 (IL-23), whose increased production is correlated with the occurrence and development of IBDs [22].

Increased concentration of the Bacteroides *B. fragilis* and *B. vulgatus* [23] was another observation in CD patients. These species demonstrate resistance to antibiotics and may turn out to be potential pathogens [24]. *Bacteroides fragilis* is a common commensal microorganism, although in inflammatory bowel diseases, its enterotoxic strain (ETBF) is dominant. It produces a zinc-dependent proinflammatory toxin and causes colitis with severe inflammation and overproduction of IL-17 [25,26]. A western diet with high amounts of saturated fatty acids (SFAs) contributes to the development of IBDs. Animal models have proven that the consumption of saturated fats from milk changes the composition of bile acids. This enables sulphate-reducing bacteria to grow, which in turn can produce large quantities of potentially mucosa-toxic hydrogen sulfide and induce helper T-cells (Th1) [27].

The characteristics of the microbiota in IBDs show that potentially proinflammatory microorganisms dominate over anti-inflammatory ones. For instance, *Faecalbacterium prausnitzii* can synthesize the microbiota anti-inflammatory molecule (MAM), which inhibits the nuclear factor kappa-light-chain-enhancer of activated B cells (NFkB) pathway [28]. The reduced presence of these microorganisms in CD causes lower synthesis of MAM, which may be associated with decreased anti-inflammatory potential. Some families of the Firmicutes phylum, including Lachnospiraceae and Ruminoccaceae, represented by *Faecalibacterium prausniztii*, are the main producers of butyrate. The decreased abundance of these species, by as much as 5 to 10 times compared to healthy controls, makes the mucosa less rich in this compound [29,30]. Short-chain fatty acids (SCFAs) are the main energy source for colonocytes, keep the intestinal mucosa healthy, and have therapeutic potential for IBDs [31]. Therefore, reduction of SCFAs in the gut may lead to the development of inflammation and increase intestinal permeability [32], which is a characteristic feature of IBD [33]. Antibiotics used in IBDs may also increase intestinal dysbiosis, thus reducing the production of SCFAs, including butyrate, which may further increase intestinal barrier integrity disorders [34]. Intestinal microorganisms can also participate in immunological reactions through their specific antigens, triggering TLRs (Toll-like receptors) and stimulating the local expansion of T lymphocytes. Specific peptides synthesized by some microorganisms contribute to the release of cytokines and the production of antibodies [35,36,37,38]. Commensal and probiotic strains can also prevent excessive inflammation by controlling the level of immunological activation in response to pathogens or other harmful antigens. These regulatory effects can be mediated by inducing the production of regulatory and anti-inflammatory cytokines (i.e., interleukin-10 (IL-10) and transforming growth factor β (TGF-β)) [39]. For instance, *Bacteroides fragilis* in combination with a polysaccharide A (PSA) molecule induces the synthesis of anti-inflammatory IL-10 using TLRs. The presence of both this bacterium and the cytokine in IBDs is reduced, which may be related to the occurrence of inflammation [40,41]. Dysbiosis occurring in CD disturbs the immune function of the microbiota, which facilitates the development of chronic inflammation.

It has been shown that the composition of the microbiome can change even within 24 h after the introduction of a nutritional intervention or a change in the diet itself [42]. For this reason, patients with diagnosed IBDs should plan their nutrition in such a way as to support the proper development of the intestinal microbiota.

## 3. Low-FODMAP Diet

The low-FODMAP diet is a very restrictive diet requiring the almost complete exclusion of substances defined as FODMAPs (Fermentable Oligosaccharides, Disaccharides, Monosaccharides, and Polyols). These include monosaccharides, disaccharides oligosaccharides, and intestinal fermenting polyols, such as fructose, lactose, or xylitol [43]. The low-FODMAP diet has been proven to minimize gastrointestinal disorders, such as abdominal pain and bloating in IBS or IBDs [44,45,46]. A low intake of saccharides fermenting in the intestines reduces the frequency of diarrhea, but it was shown that this diet does not provide similar effects in minimizing constipation [47]. Moreover, a diet with low FODMAPs can improve the quality of life of IBD patients [48].

The compounds excluded during this diet belong to the group of prebiotics, which can selectively stimulate the growth of microorganisms such as *Bifidobacterium* or *Faecalibacterium prausnitzii*. Moreover, they are substrates for the production of short-chain fatty acids [49,50]. It was noted that the relative and the absolute number of butyrate-producing bacteria decreases after a low-FODMAP dietary intervention [51]. Fructooligosaccharides may increase the abundance of *Bifidobacterium* and positively modify the immune function of dendritic intestinal mucosa cells [52]. These compounds may reduce the production of proinflammatory cytokines, e.g., IL-6, and increase that of the anti-inflammatory cytokine IL-10 [53].

A low-FODMAP diet has a significant effect on intestinal microbial composition. The total number of microorganisms may decrease up to six times after a 4-week intervention [54]. Compared to a typical Australian diet, the low FODMAP diet resulted in a decrease in the total number of intestinal bacteria, especially of *Clostridium* cluster IV, including *Faecalibacterium prausnitzii*, *Bifidobacterium*, and *Lactobacillus* [55]. A characteristic change induced by the low-FODMAP diet is the reduced number of *Bifidobacterium* [54]. In a recent study by Cox, a decreased abundance of *Bifidobacterium adolescentis* and *Bifidobacterium longum* was observed [56]. *Faecalibacterium prausnitzii*, *Ruminococcus spp.*, and *Bifidobacterium longum*, which take part in starch degradation, demonstrated to influence diet interventions in IBD [57]. Despite the limited amount of microorganisms regulating the immune response, no significant effect on inflammation markers (calprotectin or C-Reactive Protein (CRP)) was observed [56]. It has been noted that a short-term (4-week) low-FODMAP diet may even increase the levels of inflammatory markers [48].

## 4. Gluten-Free Diet

Gluten is a mixture of many proteins, mainly gliadins and glutenins. It is a form of “storage” in wheat grains. Similar compounds are found in rye (secalin), oats (avena), barley (hordein) and are collectively referred to as gluten. This compound is found in basic grains, which make up a significant part of the diet. It is contained in bread, pasta, and all other grain products, so a gluten-free diet excludes many basic energy sources and needs to be supervised by a dietitian [58,59].

A gluten-free diet is the only appropriate dietary management of celiac disease [60]. The prevalence of celiac disease or tissue transglutaminase Antibodies (anti-tTG) antibodies themselves, typical of this disease, is higher among people with diagnosed IBD than in healthy people [61]. These patients are also more likely to develop non-celiac gluten sensitivity, which causes gastrointestinal symptoms, such as abdominal pain and diarrhea after ingestion of foods containing this compound [62]. Consequently, a gluten-free diet is one of the most commonly considered elimination diets among CD patients [63].

Despite some benefits such as reducing gastrointestinal symptoms, a gluten-free diet has an impact on the composition of the intestinal microbiota [64]. Similar to the low-FODMAP diet, this dietary intervention causes a decrease in the abundance of *Bifidobacterium* microorganisms [65]. This refers mainly to *Bifidobacterium longum* species, but also to *Lactobacillus*. On the other hand, the number of *Enterobacteriaceae* and *Escherichia coli* increases [66]. A decreasing abundance of commensal bacteria combined with an increase in the number of potentially pathogenic microorganisms may have a proinflammatory and potentially negative effect on the intestinal mucosa [66]. A gluten-free diet also contributes to the decrease in *Faecalibacterium prausnitzii* abundance. As a result of unfavorable changes in the microbiota, the stimulation of host immunity is less affected, which is manifested particularly by a reduced production of IL-10 [67]. In patients with diagnosed celiac disease, a probiotic therapy minimalized gastrointestinal complaints due to changes in the intestinal microflora. In an Italian study, a 6-week Pentabiocel probiotic intake resulted in an increased amount of *Bifidobacterium*, *Staphylococcus*, and lactic acid bacteria, which reduced IBS-type symptoms in patients on a gluten-free diet. The study product consisted of a multistrain mixture containing five strains of lactic acid bacteria and bifidobacteria: *Lactobacillus casei* LMG 101/37 P-17504 (5 × 10^9^ CFU/sachet), *Lactobacillus plantarum* CECT 4528 (5 × 10^9^ CFU/sachet), *Bifidobacterium animalis* subsp. *lactis* Bi1 LMG P-17502 (10 × 10^9^ CFU/sachet), *Bifidobacterium breve* Bbr8 LMG P-17501 (10 × 10^9^ CFU/sachet), *B. breve* Bl10 LMG P-17500 (10 × 10^9^ CFU/sachet). The probiotic was given as a sachet once per day [68]. The composition and functioning of intestinal microorganisms are very sensitive to the type of food consumed. It has been shown that even a short-term introduction of a gluten-free diet may adversely affect the diversity of the microbiota by, among other mechanisms, reducing butyrate synthesis. This compound is the main source of energy for the microbiota and enables its growth [69].

## 5. Lactose-Free Diet

Lactose is a disaccharide consisting of glucose and galactose linked by a B-1→ 4 bond. The hydrolysis of this bond requires β-galactosidase, which breaks down lactose into monosaccharides, allowing these compounds to be absorbed by the intestine [70]. Low activity of this enzyme may lead to digestive symptoms similar to those of IBS, such as flatulence or diarrhea, after consumption of food containing lactose [71]. The activity of β-galactosidase is higher in children and young people and decreases with age [70]. Moreover, lactase activity is proportional to the amount of lactose in the diet [72]. It has been shown that the intestine of lactose-intolerant individuals after intake of this disaccharide produces significantly more SCFAs than the intestine of healthy patients. Increased bacterial fermentation can lead to the gastrointestinal complaints observed in lactose-intolerant people [73].

The incidence of lactose intolerance is not higher in inflammatory bowel diseases than in the general population; however, it can be observed more often in the presence of active CD. Moreover, the consumption of dairy products can reduce the risk of IBD [74,75]. Individuals suffering from gastrointestinal disorders after ingestion of lactose often exclude dairy products from their diet without attempting to introduce lactose-free dairy products. The health benefits of fermented milk include prevention of gastrointestinal infections, reduction of serum cholesterol levels, and antimutagenic activity. The consumption of fermented products by lactose-intolerant individuals and patients suffering from atherosclerosis is recommended [76,77]. However, milk-derived saturated fat can alter the composition of bile acids and allow the growth of sulfate-reducing bacteria such as *Bilophila wadsworthia*, producing toxic hydrogen sulfide [78] and worsening colitis in murine IL-10 knockout models [27].

Lactose has a prebiotic effect, inducing microbiota growth and development and promoting its diversity. It increases the abundance of probiotic *Lactobacillus* and *Bifidobacterium* species as well as of Firmicutes [79,80]. On the other hand, dairy sugar reduces the number of potentially pathogenic microorganisms, such as *Clostridium perfringens*, *Escherichia coli*, and *Proteobacteria* [79,81]. Intestinal bacteria also affect digestibility and lactose absorption. The importance of the microbiota in the proper distribution of lactose was confirmed by a study by Almeida. Lactose-intolerant patients were supplemented with a probiotic yogurt containing *L. casei Shirota* and *B. breve*. A 4-week intervention resulted in a decreased amount of exhaled hydrogen and gastrointestinal complaints after lactose intake, and this effect lasted for 3 months on average [82]. Some probiotic strains such as *Lactobacillus* and *Bifidobacterium* may also increase β-galactosidase activity [82,83,84]. Lactose supplementation is also effective in enhancing the production of SCFAs, including butyric acid [79].

Individuals with diagnosed lactose intolerance are advised not to eliminate all dairy products but to choose lactose-free products. A diet containing lactose-free milk allowed to maintain an optimal balance of the microflora in an experimental model. The mice received lactose-free or whole milk, glycomacropeptide- or soy protein (control)-supplemented diets for one month. A lactose-free milk diet was as efficient as the control diet in retaining fecal microbiota diversity in mice. Both milk diets had a significant effect on the relative abundance of health-relevant taxa (e.g., Ruminococcaceae, Lachnospiraceae). The glycomacropeptide prebiotic activity previously observed in vitro was not replicated in vivo. However, these data indicate the novel prebiotic potential of bovine milk for human nutrition [85]. Moreover, patients excluding all dairy products, including lactose-free ones, may develop nutritional deficiencies of many valuable nutrients, including calcium [86]. Lactose, synergistically with bovine milk oligosaccharides, may also influence the growth of *Bifidobacterium longum* subsp. *longum* and *Parabacteroides distasonis*, while inhibiting the growth of *Clostridium perfingers* and *Escherichia coli* [81]. Moreover, it has been shown that oligosaccharides from cow’s milk, can reduce intestinal permeability [87]. Due to limited scientific data, the European Society for Clinical Nutrition and Metabolism (ESPEN) guidelines do not recommend the routine use of lactose-free diets if no intolerance is diagnosed [88].

## 6. Ketogenic Diet

The ketogenic diet is characterized by a very low carbohydrate content, i.e., below 50 g per day, which is equal to 5–10% of the energy value of daily food [89]. This diet is suitable for patients with drug-resistant epilepsy [90].

The mechanisms underlying the protective effect of the ketogenic diet in epilepsy are not quite known. This diet causes enrichment and colonization of the gut by *Akkermansia* and *Parabacteroides*, which may have anticonvulsant effects [91]. This change in the intestinal microbiota is beneficial, but in IBDs, it results in an increase in the abundance of bacteria with potentially pro-inflammatory activity, e.g., *Parabacteroides*, inducing the progression of the disease [92]. A very-low-calorie ketogenic diet (VLCKD) causes a reduction in the total amount of intestinal microorganisms in the mouse model of autism spectrum disorders [93].

The elimination of carbohydrates leads to a decrease in prebiotic substances in the diet, which are the energy source for the growth and development of the microbiota. For this reason, the effect on intestinal bacterial diversity is similar to that of a low-FODMAP diet and is characterized by reduced numbers of *Bifidobacterium* and of the main butyrate producer, i.e., *Faecalibacterium prausnitzii* [50,94]. This will result in a reduced amount of SCFAs [95]. Butyrate contributes to the maintenance of the intestinal barrier, which is an important part of the pathomechanism of IBDs [96].

A ketogenic diet is associated with an increased intake of protein, which also influences the microbiota. This effect depends on the origin of this macronutrient. It was shown that consumption of animal protein, especially from red meat, leads to an increase in anaerobic bacteria with potential pro-inflammatory effects, such as *Bacteroides*, *Alistipes*, and *Bilophila* [97]. Furthermore, it may raise the risk of IBD development by increasing the production of hydrogen sulfide by sulphate-reducing bacteria, including *Desulfovibrio* spp. [98]. Additionally, the fermentation of animal proteins reduces the production of SCFAs, especially butyrate, and the abundance of *Bifidobacterium* in fecal microbiota [99]. On the other hand, a higher intake of plant proteins or whey proteins has the opposite effect. It was demonstrated that the consumption of whey protein and pea extracts increases the amount of *Bifidobacterium* and *Lactobacillus*, while whey further reduces the potentially pathogenic *Bacteroides fragilis* and *Clostridium perfingers* [100,101]

Another nutrient that occurs in excess in a ketogenic diet is fat, which can affect the microbiota of the digestive tract. Among all fat fractions, the development of inflammatory bowel diseases is mostly affected by trans and saturated fats [102]. It has been shown that a high-fat diet, particularly rich in SFA (saturated fatty acids), leads to an increased number of sulphate-reducing bacteria, which may raise the risk of developing intestinal inflammation [27]. Even MUFA (monounsaturated fatty acids) can cause changes in the composition of the microbiota if they occur in excess. Meanwhile, a high SFA content in the diet may reduce the overall diversity of the microbiota, including *Bifidobacterium* [103]. On the other hand, excessive amounts of PUFA (polyunsaturated fatty acids) do not affect the richness of the microbiota [103].

## 7. Specific Carbohydrate Diet (SCD)

The SCD recommends excluding complex carbohydrates in favor of more simple carbohydrates, easier to digest and absorb [104]. Originally, this diet was used for children with celiac disease (in the 1920s) [105]. In the 1980s, it gained the status of a dietary novelty for the treatment of people with inflammatory bowel diseases [106].

The regimen has been mostly promoted on the Internet by non-licensed individuals, with little supporting evidence. Indeed, the data do not strongly support the role of carbohydrates in initiating/exacerbating intestinal inflammation [107]. SCD, being a very restrictive diet, limiting the short-term consumption of most disaccharides and starches, seems to be contributing to the reduction of intestinal symptoms in IBD patients [108]. A study of the gut microbiota conducted in 2014 in eight participants aged 16–50 years with a diagnosis of CD, showed that in patients receiving the SCD diet (unlike in those receiving a diet low in plant fiber), the diversity of the microbiome increased, resulting in 134 bacteria belonging to 32 different classes. The bacterial families overrepresented in the gut ecosystem included more than 20 species of non-pathogenic bacteria of the *Clostridia* family [109]. A case study published in 2018 [110], in which SCD was used for 2 weeks in a 20-year-old patient with ulcerative colitis (UC), demonstrated that such dietary intervention significantly changed the microbiome. A decrease in the abundance of the most dominant species of fecal bacteria—*Fusobacterium ulcerans*—was observed, with a simultaneous increase in the diversity and evenness of other intestinal bacteria species. Hoffman demonstrated that carbohydrate intake might increase the counts of *Candida* and *Methanobrevibacter* archaea which utilize starch, producing simple carbohydrates. Those carbohydrates are substrates for *Prevotella* and *Ruminococcus*, producing resources consumed by *Methanobrevibacter* for methane (CH4) and carbon dioxide (CO_2_) production [111].

## 8. Paleo Diet

The Paleolithic diet was firstly described by the gastroenterologist Walter L. Voegtlin and initially named the Stone Age Diet [112]. It is based on the premise that we have not evolved sufficiently to metabolize food that is available today to humans. The creators of this diet assumed that food products that appeared as a result of the agricultural revolution (grains, milk) are harmful to human physiology because the human digestive tract is not sufficiently developed to digest such food particles. As a consequence, the Paleolithic diet is devoid of refined sugars, grains, processed vegetables and fruits, and domestic animal meats [113]. It is assumed that exposure to foods that were not present at the time of human evolution may cause modern diseases such as IBD. It seems that the microbiota of the Paleo diet can be compared to the stool microbiota of Hadza hunter–gatherers from Tanzania. Research on this unique ethnic group whose diets are based on foods available seasonally (in the rainy season, the predominant food is plant food, baobab, honey, rich in simple sugars, starch, proteins, but low in tuber fat) [114]. It was elegantly documented that in Hadza’s gut, the *Firmicutes* phylum dominates (72%), followed by *Bacteroidetes* (17%), *Proteobacteria* (6%), and *Spirochaetes* (3%). At the species level, *Prevotella* (Bacteroidetes), *Treponema* (Spirochaetes), and unclassified Bacteroidetes are noted, while *Bifidobacteria* is absent. Of note, some *Firmicutes* species, in particular *Prevotella* and *Treponema*, harbors several fiber-degrading enzymes [114].

To date, there are no data on the role of the Paleolithic diet in the treatment of IBD, except for rare and isolated, rather positive case reports [115]. However, the elevated intake of dietary fiber is positively correlated with SCFAs synthesis to further modulate GALT immune response via attenuating proinflammatory cytokines production [116] As demonstrated by Hallert et al., the increased consumption of oat bran (6 g/day) alleviated significantly the symptoms of UC [117]. Kanauchi et al. treated [118] 18 UC patients with 20–30 g/day of germinated barley foodstuff and found that *Bifidobacterium* and *Eubaterium limosum* abundance increased, which further led to improvement of bowel-related symptoms. Of note, the Paleo diet was found to increase vitamin D deficiency, thereby elevating the risk hospitalization [119].

## 9. Crohn’s Disease Exclusion Diet (CDED)

The exclusive enteral nutrition (EEN) has been described as an efficient method of inducing remission in children in comparison with steroid therapy [120]. The CD exclusion diet is a combination of enteral nutrition and whole-food restrictive diet. Oral nutrition is based on the elimination of all substances that could be allergenic or increase gastrointestinal disorders, such as milk lactose. For this reason, the diet is low in fat and animal protein and, at the same time, rich in compound carbohydrates and dietary fiber. It does not include gluten, dairy, and certain food additives such as emulsifiers, maltodextrins, carrageenan, and sulfites. In the second period, a fixed portion of whole-grain bread is allowed, as are small amounts of nuts, fruits, legumes, and vegetables. Patients with strictures continue the quantitative restriction of fruits and vegetables on an individual basis. Positive results are especially recorded in children and young adults [121,122,123,124,125,126].

In a 12-week intervention in 74 patients (mean age 14.2 ± 2.7 years), it was shown that a CDED diet in combination with enteral nutrition was better tolerated than enteral nutrition alone and was more effective in inducing remission [124]. For 6 weeks, the children received 50% of their caloric requirements from an oral diet and 50% from enteral nutrition (Modulen formula), whereas for 7–12 weeks, the industrial diet accounted for only 25% of their requirements. CRP (C-reactive protein) decreased in both groups (i.e., CDED and exclusive enteral nutrition (EEN)): in EEN, from 24 mg/dL to 4.1 mg/dL and in CDED, from 23.6 mg/dL to 5 mg/dL. Both EEN and partial enteral nutrition (PEN) affect the composition of the intestinal microbiota. For the first 6 weeks, both diets showed a similar effect on microbial activity: a reduced bacterial abundance of *Actinobacteria* and *Proteobacteria* and increased commensal *Clostridia*. However, between 6 and 12 weeks in EEN, these changes were reversed, while in CDED + PEN, the microbial composition remained similar [124]. These differences in the microbiota during the treatment may indicate a better effect of CDED + PEN compared to PEN. Another study that confirmed the effectiveness of the CDED diet is the Sigall-Boneh clinical trial [121]. Children and young adults (47 patients, mean age, 16.1 ± 5.6 years; 34 children) with active disease defined by a pediatric Crohn’s disease activity index >7.5 or a Harvey–Bradshaw index ≥4 received a 6-week structured CDED. Response and remission were achieved in 37 (78.7%) and 33 (70.2%) patients, respectively. The mean pediatric Crohn’s disease activity index decreased from 27.7 ± 9.4 to 5.4 ± 8 (*p* < 0.001), the Harvey–Bradshaw index from 6.4 ± 2.7 to 1.8 ± 2.9 (*p* < 0.001). Remission was observed in 70% of the children and 69% of the adults. Normalization of previously elevated CRP occurred in 21 of 30 (70%) patients in remission [121].

## 10. Conclusions

**Figure 2:**
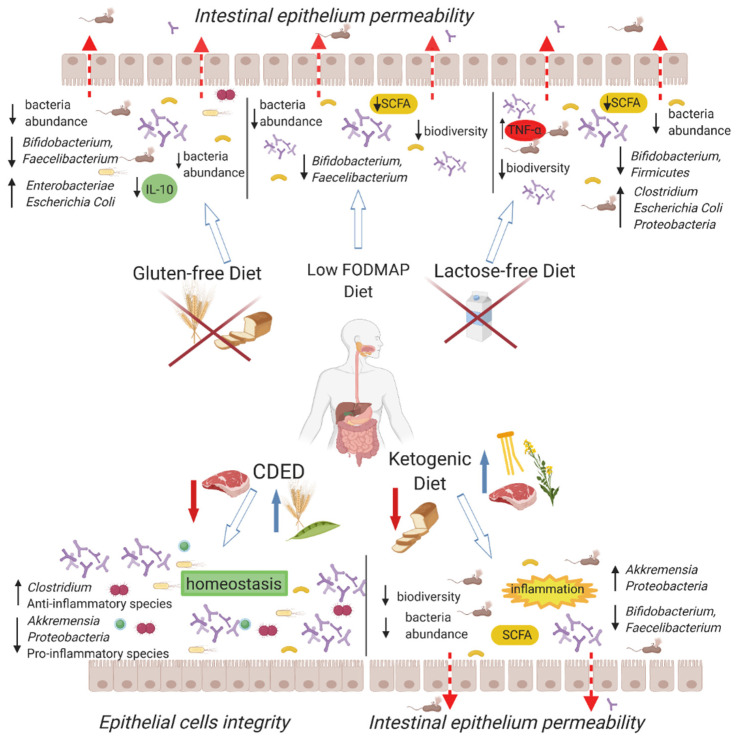
Relationship between diet and microbiome, its function, and intestinal barrier condition. Each of the indicated types of nutrition interacts with the microbiota in a characteristic way. IL-10—interleukin 10, CDED—Crohn disease exclusion diet, TNF-α–tumor necrosis factor. SCFA: Short-chain fatty acids; FODMAP: Fermentable Oligosaccharides, Disaccharides, Monosaccharides, and Polyols.

In patients suffering from CD, gut dysbiosis with low diversity of intestinal microbes is observed. Several therapeutic methods are used to alter the composition as well as the activity of the gut microbiota (Figure 2). The low-FODMAP diet may reduce the diversity of the intestinal microbiome, already impoverished by the very presence of CD. Bacteria, such as *Bifidobacterium* or *Faecalbacterium prausnitzii* are important elements, maintaining intestinal barrier integrity. A decreased abundance of those species is observed in patients with the disease. Dietary patterns eliminating prebiotic substances may exacerbate these deficiencies. Gluten-free, lactose-free, and SDC diets have similar effects on the microbiome as those of the low-FODMAP diet. The ketogenic diet, rarely used in CD, is based on animal protein, containing large amounts of saturated fats derived from meat. Excessive amounts of saturated fats lead to an increase in the number of bacteria with pro-inflammatory activity and a decrease in that of commensal ones, which may promote the development of IBDs. On the other hand, CDED provides beneficial changes in the fecal microbiome and the course of the disease, reducing the amount of bacteria with proinflammatory activity and increasing that of anti-inflammatory ones. Elimination diets appear to be effective in minimizing gastrointestinal symptoms associated with Crohn’s disease. Unfortunately, most of them can have negative effects on the microbiome and cause nutritional deficiencies. Professional dietitians with clinical experience need to be engaged for the treatment of CD patients. Nutrition recommendations should consider the effect on the quality of life and potential long-term consequences on the course of the disease.

## Figures and Tables

**Table 1:** Summary of studies in terms of the effect of nutrition on the microbiome.

Trial Name	Type of Nutrition Intervention; Population	Alternations in Microbiota
Staudacher HM, 2012	Low-FODMAP; IBS	↓ *Bifidobacterium*
Cox S.R, 2020	Low-FODMAP; Crohn disease and Colitis ulcerosa	↓ *Bifidobacterium*, in particular: *Bifidobacterium adolescentis, Bifidobacterium longum*, and *Faecalibacterium prausnitzii*
Schreiner P, 2019	Gluten-free diet, Crohn disease and Colitis ulcerosa	↓ Total amount and biodiversity
De Palma G, 2009	Gluten-free diet, healthy people	↓ *Bifidobacterium, Lactobacillus*, and *Bifidobacterium longum* ↑ *Enterobacteriaceae* and *Escherichia coli*
Marc J.B., 2016	Gluten-free diet, healthy people	↓ *Veillonellaceae, Ruminococcus bromii, Roseburia faecis* ↑ C*lostridiacea, Coriobacteiaceae*
Levine A, 2019	CDED, children with Crohn disease	↓ fecal *Proteobacteria*
Olson C.A., 2018	Ketogenic diet; children with drug-resistant epilepsy	↑ *Parabacteroides*
Lawrece D., 2014	Ketogenic diet with an increased animal protein content; healthy people	↑ *Bacteroides, Alistipes, Bilophila*
Devkota S., 2013	Ketogenic diet with an increased saturated fatty acids content; healthy people	↑ Bilophila wadsworthia
A Dubrovsky, 2018	SCD, IBD patients and healthy control	↑ *Fusobacterium ulcerans*
Schnorr SL, 2014	Paleo diet, Hadza hunter–gatherers vs. Italian control	↑ Prevotella (Bacteroidetes), *Treponema* (Spirochaetes), and unclassified *Bacteroidetes*
Halmos E., 2015	Low-FODMAP; IBS	↓ *Total amount* ↓ Cluster Clorstridium XIVa, Akkermansia municiphila, Ruminococcus

FODMAP-Fermentable Oligosaccharides, Disaccharides, Monosaccharides, and Polyols; IBD—Inflammatory bowel disease; IBS—Irritable bowel syndrome; CDED—Crohn’s Disease Exclusion Diet; SCD—Specific Carbohydrate Diet; ↓—Decreased abundance, ↑—Increased abundance.
